# Placing Public Health on the ADRD Policy Platform: Building Our Largest Dementia Infrastructure for Alzheimer’s Act

**DOI:** 10.1093/geroni/igaf122.2528

**Published:** 2025-12-31

**Authors:** Brian Kaskie, Julie Bobitt, Yogi Shah, Sarah Khasawinah

**Affiliations:** University of Iowa, Iowa City, Iowa, United States; University of Illinois at Chicago, Chicago, Illinois, United States; Broadlawns Medical Center, Des Moines, Iowa, United States; Health Policy, District of Columbia, District of Columbia, United States

## Abstract

The Building Our Largest Dementia Infrastructure for Alzheimer’s Act (BOLD Act) of 2018 placed public health onto the Alzheimer’s disease and related dementias (ADRD) public policy platform, establishing a role for state, local, territorial, and tribal public health departments to address the many challenges faced by persons living with dementia (PLwD) and those who care for them. We consider this remarkable event as another example of policy making iron triangles at both federal and state levels of government, when ADRD advocates, legislative and executive branch staff, and elected public officials who champion PLwD (i.e., a policy making iron triangle) work together to increase public investment in basic research and expand financing for care and support provided to PLwD. Upon recounting passage of the BOLD Act, we move on to highlight how 44 public health departments across the country have since been following the Centers for Disease Control and Prevention (CDC) Healthy Brain Initiative Roadmap, implementing programmatic efforts for PLwD. Referring to the BOLD Reauthorization Act of 2024, we present viable alternatives for public health leadership to sustain and expand such promising advancements bringing essential public health services to PLwD and those who care for them.

